# Prostate-Specific Antigen Velocity Predicts Surgical Outcome of Thulium Laser Enucleation of the Prostate

**DOI:** 10.3389/fmed.2021.783221

**Published:** 2022-01-03

**Authors:** Po-You Chen, Shao-Ming Chen, Horng-Heng Juang, Chen-Pang Hou, Yu-Hsiang Lin, Pei-Shan Yang, Chien-Lun Chen, Phei-Lang Chang, Kuo-Yen Lin, Ke-Hung Tsui

**Affiliations:** ^1^Department of Urology, Linkou Chang Gung Memorial Hospital, Taoyuan, Taiwan; ^2^Department of Urology, Heping Campus, Taipei City Hospital, Taipei, Taiwan; ^3^Department of Anatomy, School of Medicine, Chang Gung University, Taoyuan, Taiwan; ^4^Graduate Institute of Clinical Medical Sciences, College of Medicine, Chang Gung University, Taoyuan, Taiwan; ^5^Department of Urology, Shuang Ho Hospital, School of Medicine, College of Medical, Taipei Medical University, Taipei, Taiwan; ^6^TMU Research Center of Urology and Kidney (TMU-RCUK), Taipei Medical University, Taipei, Taiwan

**Keywords:** prostatic hyperplasia, laser, prostate-specific antigen velocity, quality of life, outcome

## Abstract

**Background:** We determined the effect of prostate-specific antigen velocity (PSAV) on the surgical outcome of thulium laser enucleation of the prostate (ThuLEP) in patients with benign prostatic hyperplasia (BPH).

**Methods:** A retrospective review was performed of prospectively collected data of patients with BPH who underwent ThuLEP at any time from 2017 to 2019. Patients who had undergone BPH surgery or had prostate cancer previously were excluded, and patients with prostate-specific antigen (PSA) > 4 ng/ml were examined through transrectal ultrasound-guided prostate biopsy to rule out prostatic malignancy. Furthermore, patients were excluded if prostatic malignancy was diagnosed during postsurgery follow-up.

**Results:** The PSA level, International Prostate Symptom Score (IPSS), and quality of life (QoL) of 27 male patients at 3 and 15 months postsurgery differed significantly from those at presurgery; the maximum flow rate (Qmax) and postvoid residual (PVR) significantly differed between 3 months postsurgery and presurgery; and 22 and 5 patients had *good* to *excellent* and *fair* to *poor* outcomes, respectively, at 15 months postsurgery. Patients were divided into two groups (*fair* and *poor* vs. *good* and *excellent* outcomes at 15 months postsurgery), which significantly differed with respect to PSAV at 3 months postsurgery (*P* = 0.04), IPSS presurgery (*P* < 0.02), surgical length (*P* = 0.01), and hospitalization duration (*P* = 0.04). In a receiver operating characteristic (ROC) analysis, the optimal cutoff value of PSAV of −0.52 ng/ml characterized effectiveness at 15 months after ThuLEP, and the area under the curve (AUC), sensitivity, and specificity were 0.82 (*P* < 0.02), 0.80, and 0.82, respectively. For PSAV < -0.52 and ≥-0.52 ng/ml, the percentages of reduction for IPSS, QoL, Qmax, and PVR were −78.6 and −71.4%, −33.3 and 0.0%, 94.4 and 40.0%, and −85.1 and −38.7%, respectively.

**Conclusions:** Postsurgical PSAV was positively correlated with surgical success, and the PSAV cutoff was −0.52 ng/ml. PSAV can, thus, be used to guide the postsurgical follow-up treatment at 3 months after BPH surgery.

## Introduction

We determined the effect of prostate-specific antigen velocity (PSAV) on the surgical outcome of thulium laser enucleation of the prostate (ThuLEP) for patients with benign prostatic hyperplasia (BPH). Before and after the BPH surgery, prostate-specific antigen (PSA) level, International Prostate Symptom Score (IPSS), quality of life (QoL), uroflowmetry, and other parameters were measured to quantify the severity of the patient's lower urinary tract symptoms (LUTS). Advanced evaluation methods include the detection of the bladder outlet obstruction (BOO) index, detrusor contractility, and presence of detrusor overactivity. Among these, PSA is used to screen for the presence of prostate malignancy before and after surgery (1–4). In addition, studies have suggested that PSA was proportional to prostate size before surgery, and PSAV was proportional to the weight of the prostate removed during surgery (5); furthermore, low PSA before surgery indicated a better outcome of prostate surgery (6, 7). Furthermore, studies have used IPSS (6), maximal uroflow rate (Qmax) (6), prostatic size (8), and BOO (9) to predict the outcome of prostate surgery. However, no study has explored the relationship between PSAV and the outcome of BPH surgery, and thus, this is investigated in this study. If the surgical outcome could be predicted within a short period postsurgery, the follow-up plan and corresponding treatment direction could be determined.

## Materials and Methods

### Population

This study reviewed prospectively collected data of patients diagnosed with BPH undergoing ThuLEP from 2017 to 2019. Patients who had BPH surgery, prostate cancer, or a diagnosis of prostate malignancy during postsurgical follow-up were excluded. Patients with PSA > 4 ng/ml before the surgery were further examined with transrectal ultrasound-guided prostate biopsy to rule out prostate malignancy. Follow-up time points were presurgery and 3 and 15 months postsurgery. The PSA level, IPSS, QoL, and uroflowmetry were recorded. The change in postsurgical PSA (PSAV) was calculated through the subtraction of the presurgery PSA value from the PSA value at 3 months postsurgery. The method for evaluating the surgical effectiveness at 15 months was based on “the criteria for efficacy of treatment in BPH” proposed by Homma et al. in 1996 (1). Patients were divided into two groups based on the effectiveness of ThuLEP at 15 months postsurgery (*excellent* and *good* vs. *fair* and *poor*), and the receiver operating characteristic (ROC) curve was used to identify the cutoff point for each clinical variable that best predicts ThuLEP effectiveness. Finally, we used bar charts to visualize the surgical results of different groups.

### Surgical Procedure

In total, 90% of the patients received spinal anesthesia, and others received general anesthesia. Surgeries in all patients were performed with the same laser machine (Cyber TM 200; Quanta System, Solbiate Olona VA, Italy) by using 800-μm caliber end-firing laser fibers. The maximum power was set at 110 W. We adopted a continuous flow 26 Ch resectoscope (Karl Storz, Tuttlingen, Germany) with a laser fiber stabilizing bridge and a classical 12-degree optic. A 26-Ch mechanical transurethral morcellator (Piranha; Wolf, Knittlingen, Germany) inserted by means of a nephroscope sheath was used in all procedures. The video source we used was a full HD Storz straight fixed camera. Physiological 0.9% sodium solution irrigation was supplied throughout the surgical procedure, with a double flow through the nephroscope sheath during prostatic morcellation. Continuous bladder irrigation was always performed for the first 12–24 postsurgical h. Foley catheter was generally removed on the third postsurgical day if macroscopic hematuria was not present.

### Design

All surgeries were performed by a single urologist in a tertiary hospital. The age, BPH duration, medical history, PSA, IPSS, QoL, and uroflowmetry readings of each patient were recorded before and after the surgery. The prostate size was measured only before the surgery. Follow-up time points were presurgery and 3 and 15 months postsurgery. The change in postsurgical PSA (PSAV) was calculated through the subtraction of the presurgical PSA value from the PSA value at 3 months postsurgery. The method for evaluating surgical effectiveness at 15 months postsurgery was based on “the criteria for efficacy of treatment in BPH” proposed by Homma et al. in 1996 (1). The IPSS, QoL, and Qmax at postsurgery were calculated separately, and the median of the three indicated the overall efficacy (Table 1). Because all clinical variables were not recorded at every time point in our study, the overall effectiveness was calculated based on the effectiveness of two individual variables. Furthermore, we documented the surgery length, hospitalization duration, incidence of hematuria and urinary retention requiring cystoscopic exploration during the follow-up period, and incidence of remedication for LUTS within 1-year postsurgery (Table 2).

**Table 1:** Standard criteria for effectiveness in various domains and overall effectiveness in BPH treatment [developed by Homma et al. (1)].

A) Symptom		
	Effectiveness	Post/pre ratio of IPSS
	*Excellent*	≤0.25
	*Good*	≤0.5
	*Fair*	≤0.75
	*Poor*	>0.75
B) Function		
	Effectiveness	Post/pre of Qmax (mL/s)
	*Excellent*	≥10
	*Good*	≥5
	*Fair*	≥2.5
	*Poor*	<2.5
C) QoL		
	Effectiveness	Pre/post of QoL index
	*Excellent*	≥4
	*Good*	3
	*Fair*	2, 1
	*Poor*	≤0
D) Overall		
	Overall effectiveness is the median of the effectiveness of 2 or 3 domains: symptom, function, or QoL.	

*IPSS, international prostate symptom score; QoL, quality of life; Qmax, maximum flow rate*.

**Table 2:** Presurgery and postsurgery clinical outcome parameters.

**Clinical variable Median (interquartile range)**	**Presurgery**	**3 months postsurgery**	**15 months postsurgery**
PSA, ng/mL	5.4 (7.0)	1.6 (1.6)^*^	1.7 (2.8)^†^
PSAV, ng/mL	—	−2.4 (4.2)	—
IPSS, score	13.0 (13.0)	2.0 (3.0)^*^	3.0 (2.0)^†^
QoL, score	3.0 (1.0)	2.0 (2.0)^*^	2.0 (2.0)^†^
Qmax, mL/s	10.0 (4.0)	13.0 (11.0)^*^	15.0 (7.0)
PVR, mL	36.0 (80.0)	9.5 (35.5)^*^	14.0 (23.0)
Effectiveness			
Good to excellent, n (%)	—	—	22 (81.48)
Fair to poor, n (%)	—	—	5 (18.52)

*PSA, prostate-specific antigen; PSAV, PSA velocity; PVR, postvoid residual; IPSS, international prostate symptom score; QoL, quality of life; Qmax, maximum flow rate. Case number = 27*.

* *Difference between values at presurgery and 3 months postsurgery were statistically significant, P < 0.017*.

† *Difference between values at presurgery and 15 months postsurgery were statistically significant, P < 0.017*.

### Data Collection

#### Statistical Analysis

The data were analyzed to determine whether to use the parametric or non-parametric method. The mean ± SD or median [interquartile range (IQR)] of the clinical values presurgery and 4 and 15 months postsurgery were used as descriptive statistics. The three time points were first compared using the Friedman's test, and each of the time points were then compared with each other. The alpha value was corrected to 0.05/3 = 0.017 by using the Bonferroni method. Patients were divided into two groups based on the effectiveness of ThuLEP at 15 months postsurgery (*excellent* and *good* vs. *fair* and *poor*), and factors that were significantly different between the groups were determined. Wilcoxon signed-rank test was used for continuous variables, and chi-square test or Fisher's exact test (for expected case number <5) was used for ordinal variables. An ROC curve was used to identify the cutoff point for each clinical variable that best predicts ThuLEP effectiveness, and the Youden index was used to determine the best cutoff point. We used bar charts to visualize the surgical results of different groups.

## Results

In total, 70 patients diagnosed with BPH successfully completed the surgery, but two of them exhibited prostatic malignancy during the follow-up period and were, hence excluded from the study. They accounted for 2.9% of the total study population. In total, 41 people were excluded from the analysis owing to a lack of PSAV data at 3 months postsurgery or lack of effectiveness at 15 months postsurgery. Finally, 27 people were included in the analysis.

The median age of the patients was 69 (9.0) years, median BPH duration was 4 (8.0) years, median prostate size presurgery was 40.7 (23.0) ml, median PSA value before surgery was 5.4 (7.0) ng/ml, and median values of IPSS, QoL score, Qmax, and postvoid residual (PVR) presurgery were 13 (13.0), 3 (1.0), 10 (4.0) ml/s, and 36 (80.0) ml respectively (Table 3). The median surgical length was 60.5 (36.0) min, and the median hospitalization duration was 2.0 (1.0) days. Zero and one patient had hematuria and had urinary retention requiring cystoscopic exploration postsurgery, respectively, and six patients received remedication for BPH within 1-year postsurgery.

**Table 3:** Factors affecting treatment effectiveness at 15 months after ThuLEP.

**Clinical variable median (interquartile range)**	**All** ***n*** **= 27**	**Effectiveness >** ***fair*** ***n*** **= 22**	**Effectiveness ≤*****fair*** ***n*** **= 5**	***P*** **value**
**Baseline characteristics**				
Patient age, year	69.0 (9.0)	69.5 (8.0)	65.0 (7.0)	0.54
BPH duration, year	4.0 (8.0)	3.5 (8.0)	5.0 (3.0)	1.00
Hypertension, *n* (%)	9 (33.3)	9 (40.9)	0 (0.0)	0.14
Diabetes mellitus, *n* (%)	5 (18.5)	4 (18.2)	1 (20.0)	1
Dyslipidemia, *n* (%)	5 (18.5)	5 (22.7)	0 (0.0)	0.55
Urinary stone, *n* (%)	5 (18.5)	5 (22.7)	0 (0.0)	0.55
**Perisurgical variables**				
Initial TRUS prostate volume, mL	40.7 (23.0)	40.7 (25.8)	41.5 (8.4)	0.97
Initial PSA, ng/mL	5.4 (7.0)	4.7 (6.5)	6.5 (11.1)	0.69
PSAV, ng/mL	−2.4 (4.2)	−2.6 (5.2)	−0.2 (4.2)	0.04^*^
Initial IPSS, score	13.0 (13.0)	13.5 (12.0)	4.0 (1.0)	<0.02^*^
Initial QoL, score	3.0 (1.0)	3.0 (1.0)	3.0 (0.0)	0.22
Initial Qmax, mL/s	10.0 (4.0)	10.0 (4.0)	10.0 (6.5)	0.75
Initial PVR, mL	36.0 (80.0)	70.5 (95.0)	31.0 (13.0)	0.21
Surgical length, minute	60.5 (36.0)	64.0 (37.0)	40.0 (17.0)	0.01^*^
Hospitalization, day	2.0 (1.0)	3.0 (1.0)	2.0 (0.0)	0.04^*^
Hematuria^†^, *n* (%)	0 (0.0)	0 (0.0)	0 (0.0)	—
Urinary retention^†^, *n* (%)	1 (3.7)	1 (4.55)	0 (0.0)	1.00
Remedication^†^, n (%)	6 (27.3)	5 (27.8)	1 (25.0)	1.00

*TRUS, transrectal ultrasound; ThuLEP, thulium laser enucleation of the prostate; PSA, prostate-specific antigen; PSAV, PSA velocity; PVR, postvoid residual; IPSS, international prostate symptom score; QoL, quality of life; Qmax, maximum flow rate; BPH: benign prostatic hyperplasia. PSAV was obtained at 3 months after the surgery.P value was the comparison between two effectiveness groups*.

* *P < 0.05*.

† *Hematuria requiring cystoscopic exploration after surgery, urinary retention requiring cystoscope exploration after surgery, or remedication of BPH medication within 1 year after surgery*.

The PSA level, IPSS, and QoL at 3 and 15 months postsurgery significantly differed from those at presurgery (Table 2). PSA decreased from 5.4 (7.0) to 1.6 (1.6) ng/ml at 3 months and to 1.7 (2.8) ng/ml at 15 months postsurgery, IPSS decreased from 13.0 (13.0) to 2.0 (3.0) at 3 months and to 3.0 (2.0) at 15 months postsurgery, and QoL decreased from 3.0 (1.0) to 2.0 (2.0) at 3 months and to 2.0 (2.0) at 15 months postsurgery. Qmax and PVR significantly differed between 3 months postsurgery and presurgery. Qmax increased from 10.0 (4.0) to 13.0 (11.0) ml/s and PVR decreased from 36.0 (80.0) to 9.5 (35.5) ml. At 15 months postsurgery, the numbers of patients for whom surgery effectiveness was good to excellent and fair to poor were 22 and 5, respectively.

Patients were divided into two groups (outcomes at 15 months postsurgery *excellent* and *good* vs. *fair* and *poor*). No significant difference was observed in age, BPH duration, chronic disease, presurgical prostate size, PSA, QoL, Qmax, PVR, number of resurgeries, and the incidence of remedication between the two groups (Table 3). Conversely, PSAV at 3 months postsurgery, IPSS presurgery, surgical length, and hospitalization duration significantly differed between the two groups. The *P*-values were 0.04, <0.02, 0.01, and 0.04, respectively. For effectiveness >*fair* and ≤ *fair*, PSAV, IPSS, surgical length, and hospitalization duration were −2.6 (5.2) and −0.2 (4.2) ng/ml, 13.5 (12.0) and 4.0 (1.0), 64.0 (37.0) and 40.0 (17.0) h, and 3.0 (1.0) and 2.0 (1.0) days, respectively.

**Figure 1:**
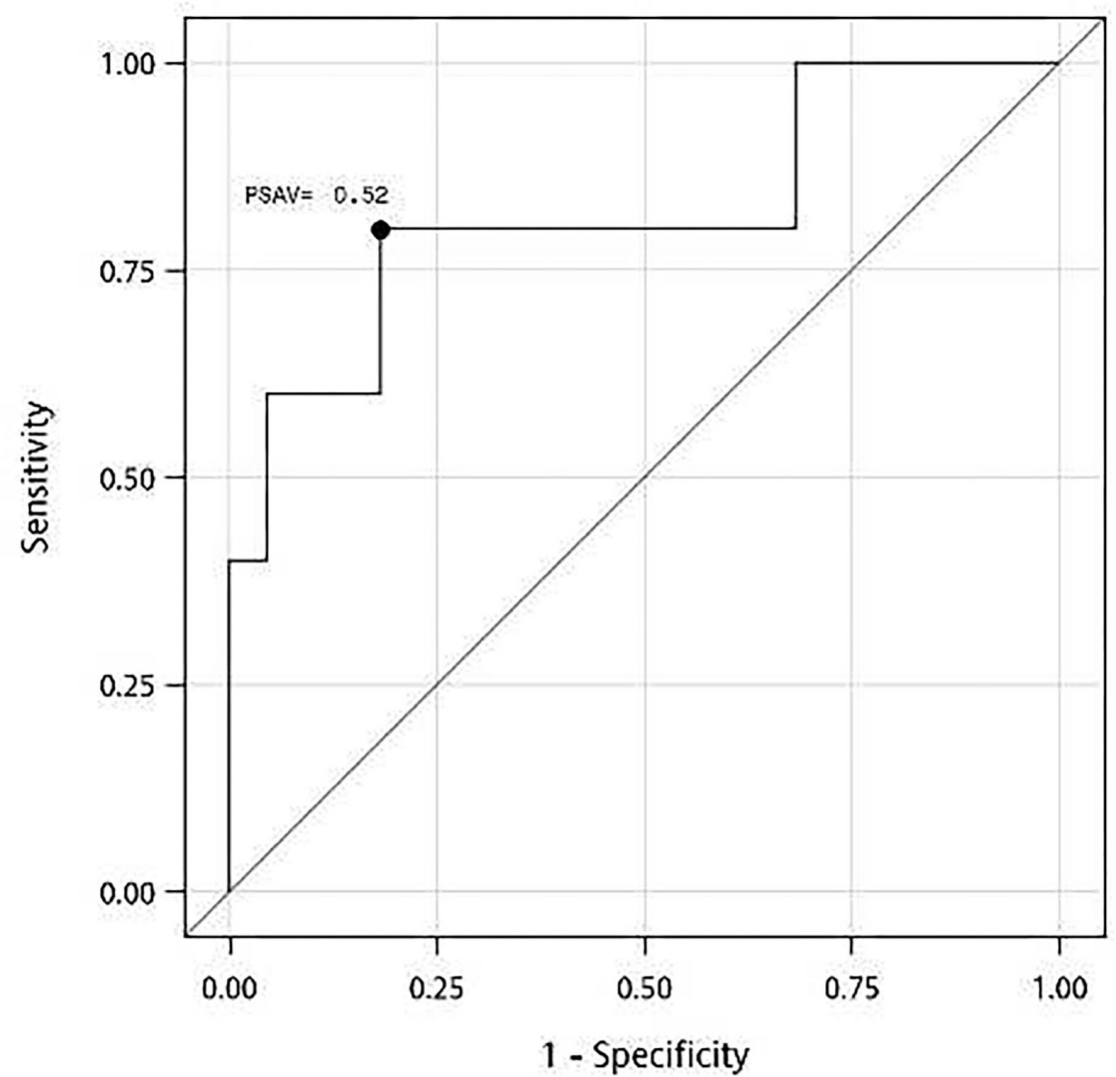
A receiver operating characteristic (ROC) curve for prostate-specific antigen velocity (PSAV) at 3 months after thulium laser enucleation of the prostate (ThuLEP) for predicting the best cutoff point for surgery effectiveness at 15 months postsurgery. Area under curve (AUC) = 0.82, with *P* < 0.02. Youden index: PSAV = −0.52 ng/ml, with sensitivity of 0.80 and specificity of 0.82.

In an ROC analysis, the optimal cutoff value of PSAV was −0.52 ng/ml for predicting the effectiveness at 15 months after ThuLEP, and the area under the curve, sensitivity, and specificity were 0.82 (*P* < 0.02), 0.80, and 0.82, respectively (Figure 1). Using the result of the ROC analysis, we divided patients with PSAV < -0.52 ng/ml into group I and those with PSAV ≥-0.52 ng/ml into group II (Table 4). No significant difference was observed in age, BPH duration, presurgical prostate size, IPSS, Qmax, PVR, surgical length, hospitalization duration, and postsurgical complications between the two groups. Presurgical PSA and QoL were slightly to significantly different between the two groups (*P* < 0.1), and the number of remedications was significantly different between the two groups. The *P*-values were 0.09, 0.07, and 0.03, respectively. For groups I and II, presurgical PSA, presurgical QoL, and number of people with remedication were 6.5 (5.8) and 2.0 (6.7) ng/ml, 3.0 (1.0) and 3.0 (0.0), and 2 and 4 people, respectively.

**Table 4:** Comparison of demographic, presurgical, and postsurgical factors of patients with different PSA velocity at 3 months after ThuLEP.

**Clinical variable** **median (interquartile range)**	**Group I PSAV < −0.52 ng/mL** ***n*** **= 19**	**Group II PSAV ≥−0.52 ng/mL** ***n*** **= 8**	***P*** **value**
**Demographic and presurgery variables**			
Patient age, year	69.0 (10.0)	68.0 (7.5)	1.00
BPH duration, year	4.0 (9.0)	4.0 (4.5)	0.73
Initial TRUS prostate volume, mL	40.8 (23.4)	39.4 (18.8)	0.68
Initial PSA, ng/mL	6.5 (5.8)	2.0 (6.7)	0.09^†^
Initial IPSS, score	14.0 (14.0)	10.5 (11.0)	0.13
Initial QoL, score	3.0 (1.0)	3.0 (0.0)	0.07^†^
Initial Qmax, mL/s	9.0 (7.0)	10.0 (4.0)	0.26
Initial PVR, mL	70.5 (82.0)	31.0 (40.0)	0.18
**Perisurgical variables**			
Surgical length, minute	63.0 (35.0)	57.5 (33.5)	0.40
Hospitalization, day	3.0 (1.0)	2.0 (0.5)	0.20
Hematuria^‡^, *n* (%)	0.0 (0.0)	0.0 (0.0)	—
Urinary retention^‡^, *n* (%)	1 (5.26)	0 (0.0)	1
Remedication^‡^, *n* (%)	2 (13.3)	4 (57.1)	0.03^*^

*TRUS, transrectal ultrasound; ThuLEP, thulium laser enucleation of the prostate; PSA, prostate-specific antigen; PSAV, PSA velocity; PVR, postvoid residual; IPSS, international prostate symptom score; QoL, quality of life; Qmax, maximum flow rate; BPH, benign prostatic hyperplasia*.

* *P < 0.05*;

† *P < 0.1*.

‡ *Hematuria requiring cystoscopic exploration postsurgery, urinary retention requiring cystoscopic exploration postsurgery; or remedication for BPH within 1 year postsurgery*.

**Figure 2:**
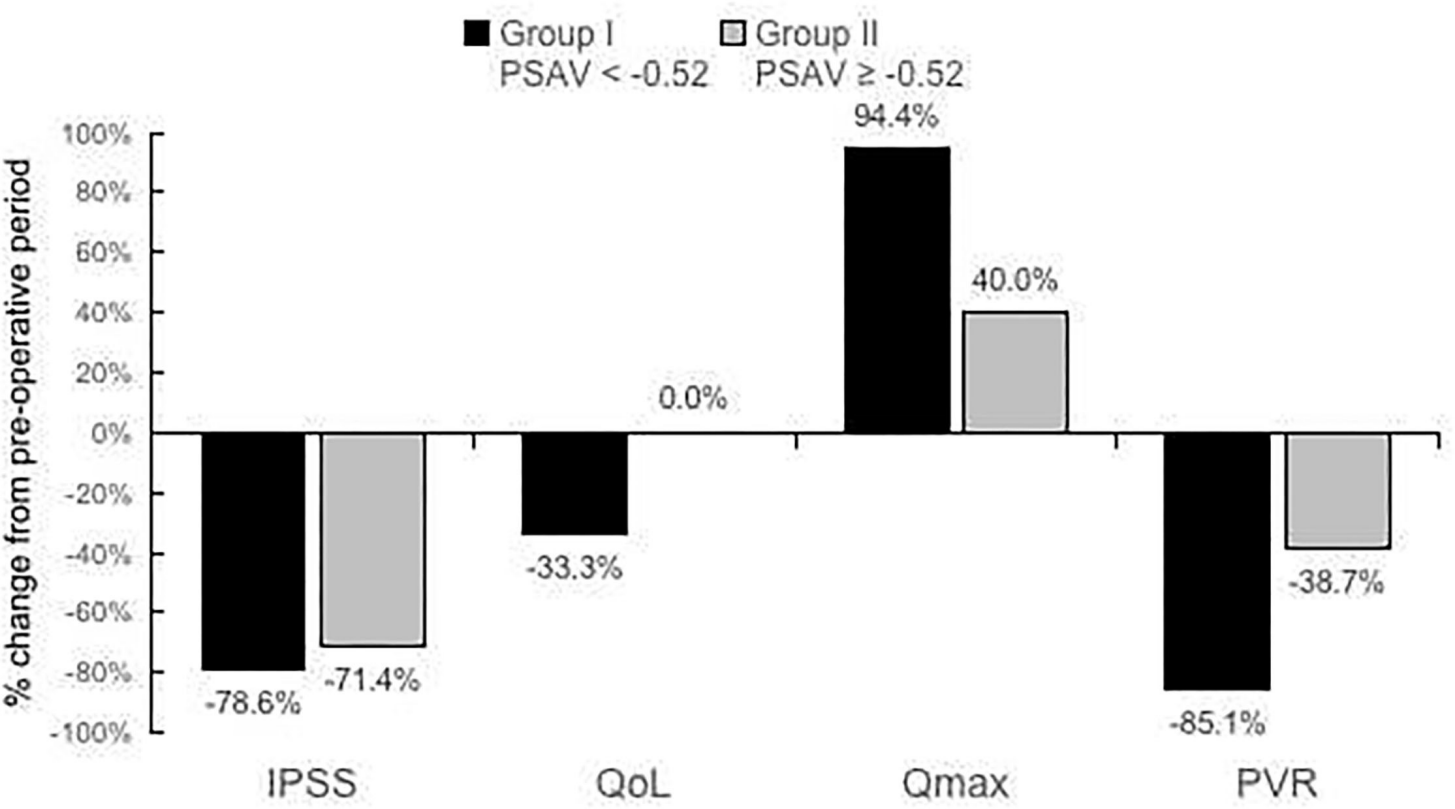
Percentage reduction of International Prostate Symptom Score (IPSS), quality of life (QoL), maximum flow rate (Qmax), and postvoid residual (PVR) at 15 months postsurgery compared with those at presurgery for PSAV < −0.52 or PSAV ≥ −0.52 ng/ml (PSAV obtained at 3 months postsurgery).

For group I and group II, the percentage reduction of IPSS, QoL, Qmax, and PVR at 15 months postsurgery and those at presurgery are presented in Figure 2 for comparison. Percentage reductions of IPSS, QoL, Qmax, and PVR were −78.6 and −71.4%, −33.3 and 0.0%, 94.4 and 40.0%, and −85.1 and −38.7%, respectively.

## Discussion

Studies have attempted to identify predictors for prostate surgery, but studies have differed in their definitions of surgical success. In 1996, Homma et al. (1) used the physician's judgment of the efficacy of surgery as the gold standard and established a prostate treatment index, such as IPSS, QoL, and Qmax. The advantage of this method was that it accounted for the changes before and after the surgery to objectively measure how much the patient's condition improved. If only the postsurgical value is considered, patients with mild symptoms will be assumed to have good surgical outcomes. However, the difficulty of this method was recording complete and large number of values before and after surgery. Both Seki et al. (10) and Ryoo et al. (9) adopted the Homma-defined system and respectively identified detrusor overactivity and BOO as factors for predicting prostate surgery. Other studies have arbitrarily set different success criteria for surgeries (6–8, 11), making it difficult to compare results between studies. Our research adopted the system defined by Homma et al., which made this study and other studies have better comparison and reproducibility.

Our study discovered that IPSS postsurgery and PSAV at 3 months postsurgery could predict surgical success. The higher the IPSS presurgery and the greater the decline in PSAV at 3 months postsurgery, the better the surgical outcome (Table 3). Reporting a similar result, Porru et al. (6) proposed that a high IPSS presurgery could lead to better surgical outcomes. Regarding Homma's criterion for surgical success, a large deviation of clinical values from the normal at presurgery might lead to increased changes postsurgery, leading to better effectiveness. In our study, a high QoL presurgery was almost significantly correlated with a decrease in PSAV (Table 4), and Porru et al. (6) and Chen et al. (11) also reported that the group with lower Qmax before surgery could have better surgical outcomes. Clinical practice has indicated that patients with severe symptoms presurgery are likely to be satisfied with the surgical outcome, and it is crucial to identify patients affected by the symptoms for surgery.

No study has mentioned whether the change in PSA could predict the outcomes of BPH surgery. One finding that supports PSAV's potential to predict the surgical outcome was that of Tinmouth et al. (5) who reported that a change in PSA after surgery is highly correlated with the reduction in prostate size. This was reasonable because previous studies have discovered that PSA and prostate size are highly and positively correlated (12). High PSAV postsurgery indicated that the volume of prostate tissue removed during surgery was high, which indicated that the patient was likely to improve from the BOO phenomenon. No study has demonstrated a correlation between the volume of prostate removed and improvement in LUTS, and this is a topic that warrants investigation. If the success of prostate surgery is positively correlated with the volume of prostate removed, then tracking PSAV postsurgery is valuable in predicting changes in prostate volume because TRUS of the prostate is an invasive procedure.

In this study, we found that an increased PSA presurgery was almost significantly correlated with a decreased PSAV during surgery (Table 4). This result contradicted the results of previous studies. Te et al. (7) observed that presurgical PSA <6 ng/ml and a small prostate volume were correlated with improved surgical outcome, and Elkoushy et al. (8) also reported that participants requiring reoperation had a large prostate volume. Both these studies have reported that photoselective vaporization prostatectomy (PVP) and transurethral incision of the prostate (TUIP) are more commonly used for the small prostate. (13) Therefore, the amount of prostate resection might be insufficient for a large prostate, leading to worse surgical outcomes. This was demonstrated in the study by Te, where PSA decline was 17% at 3 years of follow-up, whereas PSA decline in transurethral resection of prostate and holmium laser enucleation of prostate were both >70%. (5) Therefore, PVP and TUIP were designed for a small prostate. If the prostate could be hollowed out, the large PSA and prostate size presurgery might not lead to worse surgical outcome (9). However, prostate size and PSA presurgery were not good predictors of successful surgery because prostate resection volume for each surgery was different; hence, PSAV or prostatic volume change may be useful to assess the completeness of prostate resection.

With a decrease in PSA, LUTS and uroflowmetry improved (Figure 2), and the rate of remedication significantly decreased (Table 4). Notably, the cutoff value of PSAV = −0.52 ng/ml analyzed using the ROC curve was relatively small compared with PSA value before the surgery (5). Hence, if the change in PSA postsurgery was too small, it was possibly because the prostate was not thoroughly resected during the surgery.

Although PSAV and change in LUTS were found to be correlated in our study, no correlation has been observed between PSA or prostate volume and IPSS or QoL in the literature (12, 14). This was probably because the peripheral region of the prostate occupies much volume despite having no direct influence on the production of BOO and LUTS; thus, prostate size was not observed to be correlated with LUTS. However, the resected prostate field was directly related with BOO, prostate size change and LUTS or BOO change might be correlated.

This study was limited by its retrospective nature. Moreover, in an attempt to meet all of Homma's criteria, the case number selected was relatively small. Furthermore, the follow-up period was short. However, with a long follow-up period, the problem of incomplete data would be greater due to loss of follow-up. Patients with BPH were prone to losing track of follow-up postsurgery.

Nevertheless, as a pioneer study for PSAV, compared with previous studies that used presurgical clinical predictors, this study proposed PSAV for evaluating surgery execution and predicting surgery effectiveness. PSAV determination is cheap, non-invasive, and easily executable in clinical settings. Furthermore, this study used Homma's criterion for BPH treatment, which increased the comparability and reproducibility of this study with other studies and facilitated further corresponded research.

## Conclusion

Prostate-specific antigen velocity postsurgery was positively correlated with surgical success, and the cutoff point of PSAV was −0.52 ng/ml. At 3 months after the BPH surgery, postsurgery follow-up plan and treatment may be determined based on PSAV, which predicts surgical success.

## Data Availability Statement

The raw data supporting the conclusions of this article will be made available by the authors without undue reservation.

## Ethics Statement

The studies involving human participants were reviewed and approved by Chang Gung Memorial hospital. The patients/participants provided their written informed consent to participate in this study.

## Author Contributions

All authors contributed significantly to the development of this article. P-YC, K-HT, Y-HL, C-PH, H-HJ, S-MC, C-LC, P-SY, and P-LC: conceptualization. S-MC and C-PH: conception and design. K-HT, Y-HL, S-MC, C-LC, and P-LC: enrollment of patients and acquisition of data. C-PH, Y-HL, S-MC, and H-HJ: drafting of the manuscript. C-LC and H-HJ: statistical analysis. K-HT, Y-HL, C-PH, P-SY, and C-LC: analysis and interpretation of data.

## Funding

This study was funded by the Chang Gung Memorial Hospital, Grant: CMRPG3H1321-2, CMRPG3K0821, CLRPG3K0021. Taiwan National Science Foundation grants NSC 110-2314-B-038-151-MY3. The authors thank TMU Academic Editing for editing this manuscript.

## Conflict of Interest

The authors declare that the research was conducted in the absence of any commercial or financial relationships that could be construed as a potential conflict of interest.

## Publisher's Note

All claims expressed in this article are solely those of the authors and do not necessarily represent those of their affiliated organizations, or those of the publisher, the editors and the reviewers. Any product that may be evaluated in this article, or claim that may be made by its manufacturer, is not guaranteed or endorsed by the publisher.
